# Brain endothelial PTPRO drives LPS-induced metabolic reprogramming and neuroinflammation in sepsis-associated encephalopathy

**DOI:** 10.1186/s12974-026-03790-7

**Published:** 2026-03-29

**Authors:** Fengjiao Wu, Haitao Tang, Haoxuan Jia, Chang Li, Yuwei Song, Jiwei Hu, He Cao, Benben Zhang, Xiaoyu Zhang, Hairui Jiang, Zhengyan Pan, Wusheng Zhou, Zongming Gou, Tianzhen Li, Tianbao Li, Hongtao Wang, Huaxue Wang, Zhongqing Qian, Ting Wang

**Affiliations:** 1 Interdisciplinary Eye Research Institute (EYE-X Institute), Anhui Provincial Key Laboratory of Immunology in Chronic Diseases, Anhui Province Key Laboratory of Basic and Translational Research of Inflammation-related Diseases, Anhui Provincial Key Laboratory of Infection and Immunology, Department of Immunology, School of Basic Medical Sciences, Bengbu Medical University, Bengbu, 233030 China; 2 Departments of Critical Care Medicine, The First Affiliated Hospital of Bengbu Medical University, Bengbu, 233030 China; 3 Center for Translational Science, Florida International University, 11350 SW Village Parkway, Port St. Lucie, FL 34987 USA

**Keywords:** PTPRO, Sepsis-associated encephalopathy, Brain endothelial cells, Glycolysis, HIF-1α, Blood-brain barrier, Neuroinflammation

## Abstract

**Background:**

Sepsis-associated encephalopathy (SAE) is characterized by profound neuroinflammation and blood–brain barrier (BBB) disruption, yet the molecular mechanisms linking endothelial metabolic reprogramming to neuroinflammatory responses remain poorly defined. Protein tyrosine phosphatase receptor type O (PTPRO) has been implicated in endothelial signaling; however, its role in metabolic and inflammatory dysregulation during SAE is unknown.

**Methods:**

We analyzed transcriptomic responses to lipopolysaccharide (LPS) challenge in control and PTPRO-silenced brain microvascular endothelial bEND.3 cells to define the impact of PTPRO on endothelial gene expression under septic stress. Endothelial cells with PTPRO overexpression, siRNA-mediated knockdown, or pharmacologic inhibition were assessed for glycolytic activity, glucose uptake, HIF-1α signaling, BBB integrity, and inflammatory responses following LPS exposure. LPS-induced neuroinflammation models were established in wild-type, systemic PTPRO knockout (PTPRO⁻/⁻), and endothelial-specific PTPRO conditional knockout (E-cKO) mice, and disease severity was systematically evaluated.

**Results:**

We provide the first evidence that PTPRO regulates LPS-induced glycolytic pathways at the transcriptomic level. PTPRO silencing or inhibition in bEND.3 cells significantly attenuated LPS-induced glycolytic activation, HIF-1α accumulation, glucose transporter translocation, and lactate production. In vivo, LPS-induced neuroinflammation was associated with enhanced glycolysis, increased HIF-1α expression, BBB disruption, and elevated neuroinflammatory cytokine production; these effects were markedly reduced in both systemic and conditional endothelial-specific PTPRO-deficient mice, with preservation of BBB integrity and decreased neutrophil infiltration. Mechanistically, PTPRO modulated ErbB2 phosphorylation at multiple tyrosine residues and activated AKT–mTOR signaling under inflammatory conditions, linking PTPRO to endothelial glycolytic reprogramming and inflammatory activation. Pharmacologic inhibition of HIF-1α or glycolysis suppressed pro-inflammatory cytokine production, indicating that PTPRO promotes endothelial inflammation through a HIF-1α–dependent glycolytic pathway.

**Conclusions:**

PTPRO drives endothelial glycolytic reprogramming and neuroinflammation in LPS-induced neuroinflammation by regulating HIF-1α signaling through the ErbB2–AKT–mTOR axis. Targeting PTPRO confers metabolic, neurovascular, and anti-inflammatory protection, highlighting PTPRO as a promising therapeutic target for SAE.

**Graphical abstract:**

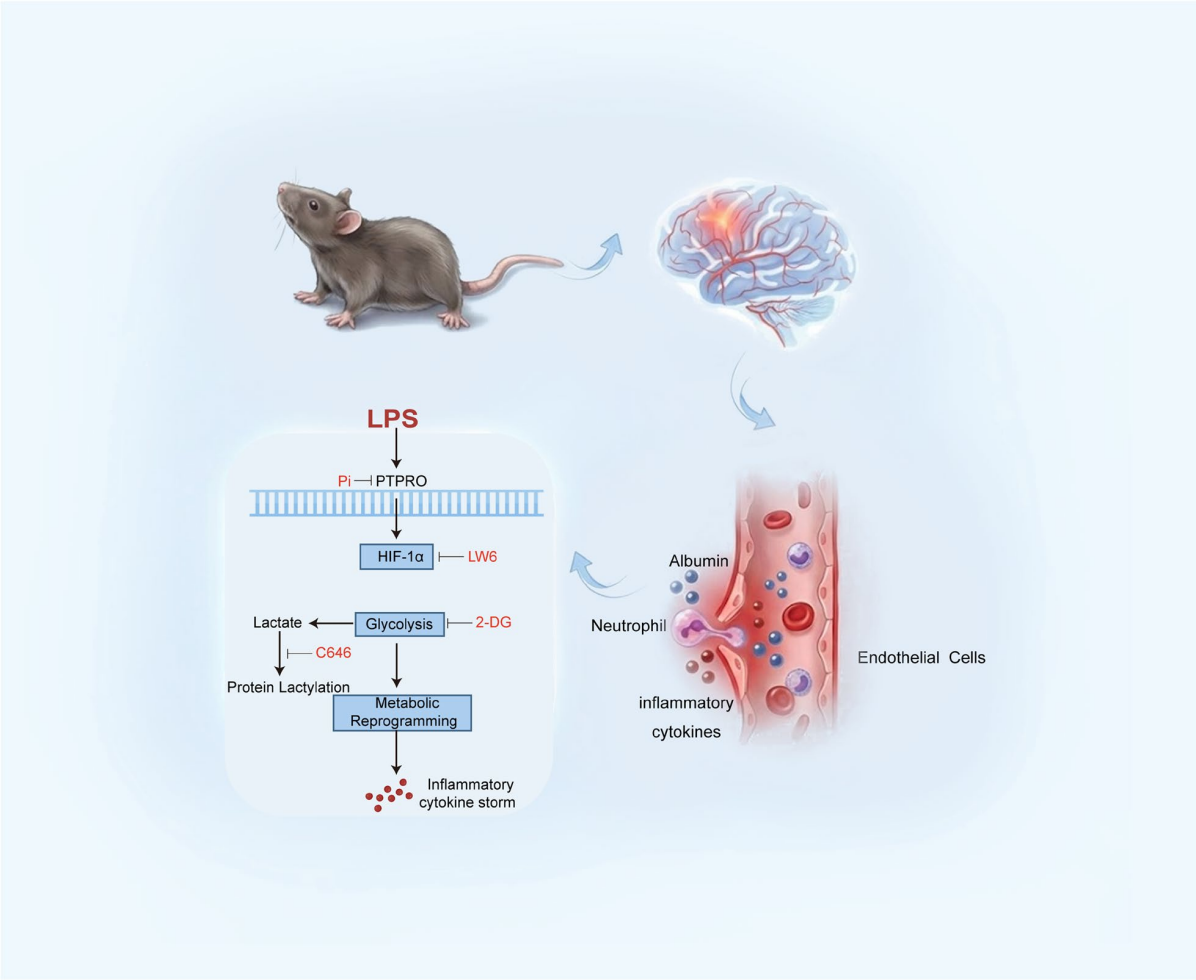

**Supplementary Information:**

The online version contains supplementary material available at 10.1186/s12974-026-03790-7.

## Introduction

Sepsis-associated encephalopathy (SAE) is a frequent and severe complication of systemic infection, characterized by blood–brain barrier (BBB) disruption, neuroinflammatory dysregulation, and acute cognitive impairment [1]. The pathophysiology of SAE involves complex interactions among endothelial dysfunction, metabolic reprogramming, and immune activation within the central nervous system (CNS) [1]. Cerebral microvascular endothelial cells are central to maintaining BBB integrity and modulating neuroinflammation, and their functional impairment contributes directly to SAE progression [2]. The BBB is formed primarily by brain endothelial cells that tightly regulate paracellular and transcellular transport to protect the CNS from circulating insults [3]. During sepsis, endothelial cells are directly exposed to circulating pathogen-associated molecular patterns (PAMPs) such as lipopolysaccharide (LPS), leading to endothelial activation, barrier dysfunction, and leukocyte infiltration into the brain parenchyma [4]. Beyond structural impairment, the brain endothelium undergoes profound metabolic adaptations in response to inflammatory stress [5]. Emerging studies suggest that endothelial metabolic reprogramming actively shapes inflammatory signaling and vascular permeability [5]. However, how metabolic changes in brain endothelial cells contribute to neuroinflammation during SAE remains poorly defined.

Recent studies have highlighted that endothelial metabolic reprogramming, particularly a shift from oxidative phosphorylation toward glycolysis, underlies inflammatory activation and BBB breakdown during sepsis [6, 7]. Hypoxia-inducible factor 1α (HIF-1α) acts as a master transcriptional regulator of glycolytic genes, linking metabolic adaptation to inflammatory signaling in endothelial cells [8]. Enhanced glycolytic flux not only fuels cellular energy demand under stress but also promotes lactate accumulation, which further amplifies pro-inflammatory responses [9, 10]. Despite these general concepts, the upstream molecular regulators that coordinate endothelial glycolysis and inflammatory activation in SAE remain poorly defined.

Protein tyrosine phosphatase receptor type O (PTPRO) is a receptor-type tyrosine phosphatase implicated in the regulation of receptor tyrosine kinase signaling, cellular metabolism, and inflammation in various tissues [11]. Notably, PTPRO can modulate the phosphorylation of ErbB2 [12], thereby influencing downstream PI3K/AKT/mTOR signaling pathways, which are critical for glycolytic programming and inflammatory responses. Our previous studies suggest PTPRO is highly inducible during septic conditions by LPS [13], however, the role of PTPRO in cerebrovascular endothelial cells and its contribution to glycolytic reprogramming during the pathogenesis of SAE remain largely unknown.

In the present study, our non-biased bioinformatics analysis first-time suggested PTPRO drives a selective impact on cellular glycolysis, potentially via HIF-1α-dependent pathways. We further employed LPS-induced neuroinflammation mouse models, endothelial-specific PTPRO knockout mice, and in vitro brain endothelial cell models to investigate the mechanistic role of PTPRO in endothelial metabolism and neuroinflammation. We demonstrate that PTPRO promotes HIF-1α-dependent glycolytic reprogramming, lactate accumulation, and pro-inflammatory cytokine production via ErbB2–AKT–mTOR signaling. Furthermore, endothelial-specific deletion or pharmacological inhibition of PTPRO protects against BBB disruption and attenuates neuroinflammatory responses during LPS-induced neuroinflammation.

Together, this study uncovers a previously unrecognized endothelial metabolic checkpoint that integrates inflammatory signaling with glycolytic reprogramming and BBB dysfunction in SAE. Targeting PTPRO-mediated metabolic pathways may therefore represent a novel therapeutic strategy for mitigating sepsis-associated brain injury.

## Materials and methods

### Antibodies

All antibodies were obtained from commercial vendors, and detailed information including catalog numbers and working concentrations (or dilution ratios) is provided in Supplementary Table 1.

### Animals

All C57BL/6J mice aged 7–8 weeks were purchased from Gempharmatech (Nanjing, Jiangsu, China). PTPRO^−/−^ mice (C57BL/6JGpt-Ptproem8Cd96946in1/Gpt, Strain #T014090), PTPRO^flox/flox^ mice (C57BL/6JGpt-Ptproem1Cflox/Gpt, Strain #T013208), Tek-iCre mice (Strain #T003764) and Elane-iCre (Strain # T006195) were purchased from GemPharmatech. PTPRO Endothelium conditional knockout (E-cKO) mice were generated by crossing PTPRO^flox/flox^ mice with Tek-Cre heterozygous mice, as a homozygous Tek-Cre mouse is lethal. This breeding produced both Tek-Cre^(+)^/PTPRO^flox/flox^ and Tek-Cre^(−)^/PTPRO^flox/flox^ offspring. The Tek-Cre^(−)^/PTPRO^flox/flox^ mice, lacking Cre expression but retaining the floxed allele, served as controls. Tek-Cre^(+)^/PTPRO^flox/flox^ mice are noted as E-cKO mice. PTPRO neutrophil-specific conditional knockout (N-cKO) mice were generated by crossing PTPRO^^flox/flox^ mice with Elane-iCre mice. This breeding strategy yielded Elane-iCre(+)/PTPRO^^flox/flox^ mice and Elane-iCre(−)/PTPRO^^flox/flox^ littermates. Elane-iCre(−)/PTPRO^^flox/flox^ mice were used as control mice, whereas Elane-iCre(+)/PTPRO^^flox/flox^ mice were designated as N-cKO mice. Mice were housed in an environment under a 12-h light/dark cycle at around 20–24 °C with 45–65% humidity with ad libitum access to food and water. All animal programs were conducted in accordance with guidelines and laws, and were approved by the animal ethics committees of Bengbu Medical University.

### Murine LPS-induced neuroinflammation model

LPS-induced neuroinflammation model was established by intraperitoneal injection with LPS as previously described [14]. Briefly, male C57BL/6 mice (10–12 weeks old, 22–25 g) were intraperitoneally injected with lipopolysaccharide (LPS, 10 mg/kg, Sigma-Aldrich, St. Louis, MO) dissolved in 200 µL of PBS. Control mice received an equivalent volume of PBS alone. To inhibit PTPRO in vivo, mice were intraperitoneally injected with PTPRO inhibitor 30 min prior to LPS administration. Control mice received an equivalent volume of vehicle (0.1% DMSO; Sigma-Aldrich) diluted in PBS. Mice were euthanized 22 h after LPS injection, and brains were harvested for downstream analyses.

### Cell culture

bEND.3 cells (ATCC, Manassas, VA) were cultured under ATCC-recommended conditions: high-glucose DMEM supplemented with 10% FBS at 37 °C and 5% CO_2_ in a humidified incubator. For functional assays, cells were maintained in endothelial growth medium (Lonza, Morristown, NJ).

### Lentiviral transduction and stable overexpression

bEND.3 cells were cultured to approximately 70%–80% confluence before transduction. Lentiviral particles encoding PTPRO or control vectors were added to the culture medium together with Polybrene (Sigma-Aldrich) to enhance viral entry. Before stable transduction, a puromycin kill-curve was performed in bEND.3 cells using 0.5, 1, 1.5, 2, 3, and 4 µg/mL puromycin, and the lowest concentration that killed > 90% of non-transduced cells within 48 h was used as the effective selection concentration. Cells were infected under optimized MOI conditions, and transduction efficiency was evaluated 72 h later. The condition yielding approximately 80% transduction efficiency with good cell viability was selected for subsequent experiments. Puromycin selection was then applied to eliminate non-transduced cells and establish stable cell populations. After selection, stably transduced cells were expanded and used for downstream functional and biochemical assays. During subsequent culture, puromycin was applied periodically as maintenance selection. Stable overexpression was verified by Western blot analysis of PTPRO expression before use in downstream experiment.

### siRNA-mediated gene silencing

PTPRO-specific siRNA (sense: GGUCAUACCGAAUCUCAAUTT; antisense: AUUGACAUUCGGUAUGAGCTT) and corresponding control siRNA were obtained from GenePharma (Shanghai, China). Cells were seeded into 12-well plates and grown to approximately 60% confluence to ensure optimal transfection efficiency while maintaining cell health. Transfection was performed using Lipofectamine 3000 reagent (Cat# L3000001; Invitrogen, Carlsbad, CA, USA) according to the manufacturer’s protocol, which included complex formation of siRNA with Lipofectamine in serum-free medium followed by addition to cells. After 6 h, the medium was replaced with complete endothelial growth medium (Lonza) to minimize cytotoxicity. Cells were harvested 48 h post-transfection to assess knockdown efficiency and downstream signaling effects.

### RNA sequencing

**Fig. 1:**
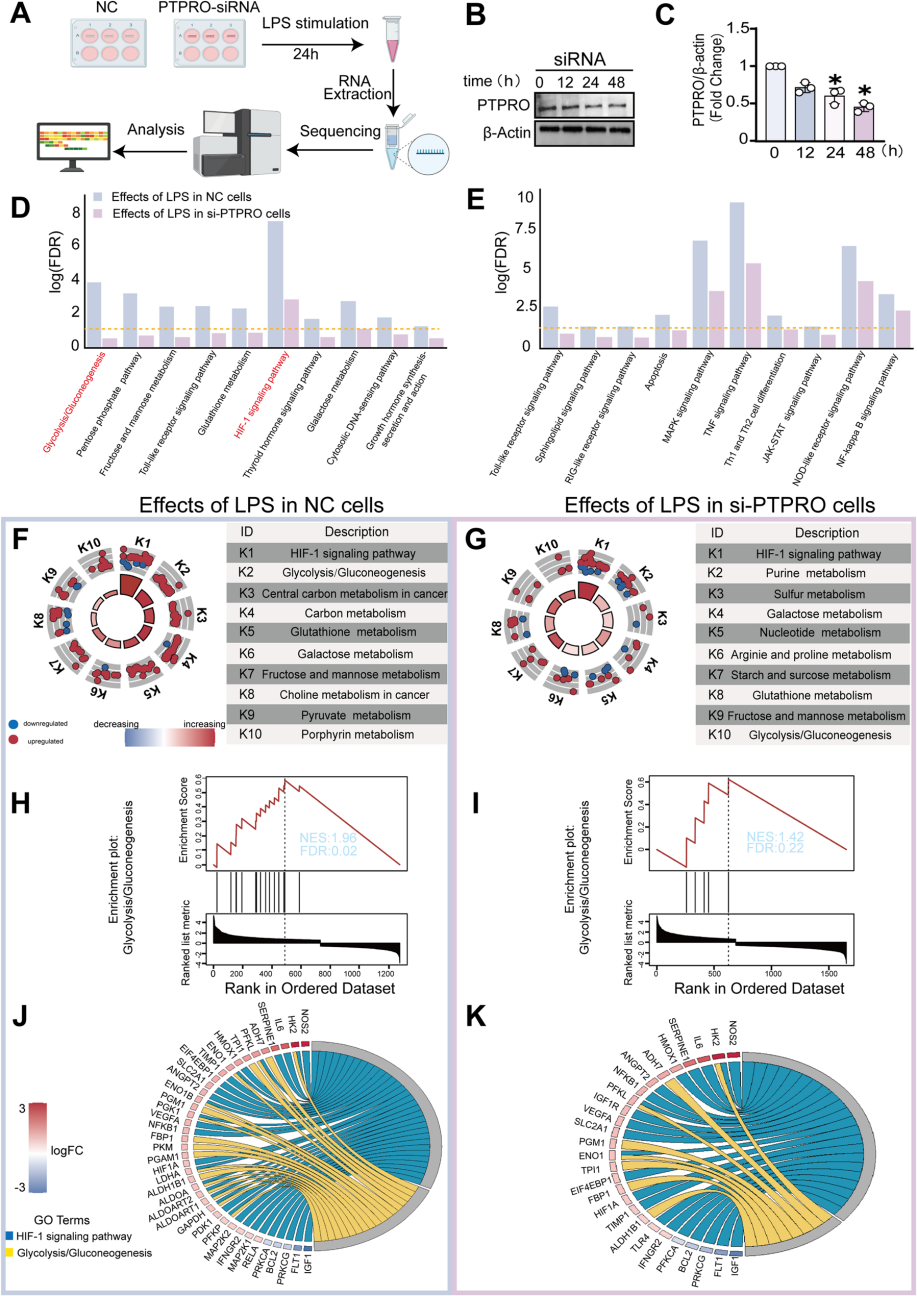
PTPRO silencing reshaped the LPS-induced transcriptional landscape and attenuated glycolytic pathway activation. **A** Schematic illustration of the experimental workflow for transcriptomic profiling of negative control (NC) and PTPRO-silenced cells, in which bEND.3 cells were transfected with control or PTPRO siRNAs for 24 h followed by LPS stimulation for an additional 24 h prior to RNA collection. **B**–**C** Immunoblot analysis of PTPRO protein expression in NC and PTPRO-silenced cells (n = 4). **D**–**E** KEGG pathway enrichment analysis based on differentially expressed genes identified from RNA sequencing. **F**–**G** Global pathway activity scoring analysis comparing metabolic pathway activities between NC and PTPRO-silenced cells. **H**–**I** Gene Set Enrichment Analysis (GSEA) of predefined glycolysis-related gene sets in NC and PTPRO-silenced cells. **J**–**K** Circos plots depicting gene expression profiles associated with glycolysis/gluconeogenesis and HIF-1 signaling pathways. Data are presented as mean ± SD. * *p* < 0.05 vs. NC

Four experimental groups of endothelial cells (*n* = 3 per group) were prepared for RNA sequencing (RNA-seq), including control, LPS, si-PTPRO, and si-PTPRO + LPS. Total RNA was extracted from cultured endothelial cells using the RNeasy Plus Mini Kit (QIAGEN, Düsseldorf, Germany) according to the manufacturer’s instructions. RNA quality and integrity were evaluated prior to library preparation. cDNA libraries were constructed and sequenced on an Illumina HiSeq platform by LC-Bio Technology. Raw sequencing reads were subjected to quality control using FastQC (v0.11.9), and adaptor sequences and low-quality bases were removed using Trimmomatic (v0.39). Clean reads were aligned to the mouse reference genome (GRCm38/mm10) using HISAT2. Gene-level read counts were generated using HTSeq, and gene expression levels were normalized as fragments per kilobase of transcript per million mapped reads (FPKM). Differentially expressed genes (DEGs) between experimental groups were identified using the limma package in R [15], with Benjamini–Hochberg correction applied to control the false discovery rate. Genes with |log2 fold change| > 1 and adjusted *P* < 0.05 were considered significantly differentially expressed. Pathway enrichment analysis was performed using the DAVID (v6.8) database with KEGG pathway annotations. Enriched pathways were ranked based on enrichment significance and differences in enrichment P values between NC and siPTPRO conditions following LPS stimulation. Gene Set Enrichment Analysis (GSEA) was performed using KEGG pathway gene sets to evaluate enrichment of glycolysis-related and HIF-1 signaling pathways based on ranked gene expression changes between experimental groups. To visualize gene–pathway relationships (Fig. 1J–K). Circos plots were generated in R using the circlize package, integrating KEGG pathway annotations with the differentially expressed genes identified in the RNA-seq analysis.

### Glucose uptake assay

Cellular glucose uptake was measured using a Glucose Uptake Assay Kit (Abcam, Waltham, MA, USA, Cat# ab136955). bEND.3 cells were plated in confocal culture dishes and incubated overnight in endothelial growth medium (Lonza). Before probe loading, cells were washed twice with prewarmed glucose-free, serum-free medium and incubated in glucose-free, serum-free medium for 15 min at 37 °C. The glucose uptake probe working solution was prepared freshly and pre-equilibrated to 37 °C, then added to the cells for 15 min at 37 °C in 5% CO₂. Subsequently, quenching buffer prewarmed to 37 °C was added to suppress extracellular probe fluorescence, and images were captured within 10 min using a Nikon AXR NSPARC confocal microscope. Fluorescence was recorded at Ex/Em = 488/520 nm. All glucose metabolism experiments were performed under identical culture conditions without manipulation of extracellular glucose levels. Thus, osmotic controls (e.g., mannitol or L-glucose) were not included, as LPS stimulation was the only variable.

### Determination of glucose and L-lactic acid levels

Brain tissues or cells were homogenized and then centrifuged at 12,000 rpm for 40 min at 4 °C. The supernatants were collected, and the total protein concentrations were measured using a BCA assay kit. Glucose and L-lactate levels in the cell lysate were subsequently measured using commercial assay kits (Glucose assay kit, Lactate dehydrogenase assay kit, Cat# F006-1-1 and #A020-2-2, Jiancheng Bioengineering Institute, Nanjing, China; L-lactate assay kit, Cat# E-BC-K044-M, Elabscience, Wuhan, China) according to the manufacturers’ instructions.

### Measurement of ATP levels

Brain tissues were weighed and homogenized in 1 mL of deionized water while kept on ice. The homogenate was then heated at 100 °C for 5 min to extract metabolites, followed by centrifugation at 8,000 × g for 15 min at 4 °C. The supernatants were collected, and ATP levels were measured using a Micro ATP Content Assay Kit (Cat#KTB1016; Abbkine, Wuhan, China) according to the manufacturer’s instructions.

### Glycolysis stress test (ECAR measurement)

Extracellular acidification rate (ECAR) was measured using a Seahorse XF24 Analyzer (Agilent/Seahorse Bioscience, Santa Clara, CA). Briefly, bEND.3 cells were seeded at a density of 2 × 10^4^ cells per well in XF24 cell culture microplates and allowed to adhere overnight. Prior to the assay, cells were washed and incubated in XF DMEM assay medium (pH 7.4) supplemented with 1 mM sodium pyruvate and 1 mM L-glutamine. For ECAR measurements, glucose, oligomycin, and 2-deoxy-D-glucose (2-DG) were sequentially injected through the designated ports at final concentrations of 10 mM glucose, 1 μM oligomycin, and 50 mM 2-DG, respectively. ECAR was recorded in real time according to the manufacturer’s instructions, and data were analyzed using Seahorse Wave software.

### RNA extraction and quantitative real-time RT-PCR

For brain tissue samples, total RNA was extracted from whole-brain homogenates using TRIzol reagent according to the manufacturer’s instructions (Invitrogen), followed by phenol/chloroform extraction. Total RNA from cultured cells was extracted using the same procedure. Total RNA was reverse transcribed into cDNA using Transcript All-in-One First-Strand cDNA Synthesis SuperMix (TransGen Biotech, Beijing, China). Quantitative real-time PCR was performed using ChamQ Universal SYBR qPCR Master Mix (Vazyme, Nanjing, China; Q711-02) on a Roche LightCycler 480 real-time PCR system. β-Actin was used as the internal control. Relative mRNA expression levels were calculated using the 2^^−ΔΔCt^ method. The primers used in this study were designed and synthesized by General Biol (Anhui, China), and all primer sequences are listed in Supplementary Table 2. Primer specificity was confirmed by melting curve analysis showing a single peak.

### Western blot

The total protein, surface membrane protein, and cytoplasmic protein samples were extracted from whole-brain tissues of C57BL/6 mice or cells for immunoblotting analysis. For total protein extraction, tissues and cells were homogenized in 1000 µL of lysis solution composed of 40% SDS, 60% RIPA buffer, and 1% protease inhibitor (Cat#539131; Millipore, MA, USA) per 100 mg of brain tissue. The homogenate was then centrifuged at 13,000 rpm for 30 min, and the supernatants (containing cytosolic and membrane fractions) were collected. Extraction of surface and cytoplasmic proteins was conducted using the Surface and Cytoplasmic Protein Reagent Kit (Cat#P0033; Beyotime, Shanghai, China) according to the manufacturer’s instructions. Protein concentrations were measured spectrophotometrically using the BCA kit (Cat#A50668; Thermo-Fisher SCIENTIFIC, Waltham, MA, USA). Protein samples were fractionated by SDS-polyacrylamide gel electrophoresis and then transferred to polyvinylidene difluoride (PVDF) membranes (Cat#IPVH85R, Sigma-Aldrich). The membrane was incubated with blocking buffer (5% BSA in TBST buffer) for 1 h, and in primary antibody (in blocking buffer) overnight at 4 °C. The membrane was then washed three times with TBST buffer, and incubated in secondary antibody at room temperature for 1 h. The target protein was visualized then by using electrochemiluminescence (ECL) reagent (Cat#34577; Thermo-Fisher) with a Bio-Rad ChemiDoc system (Hercules, CA, USA). The band intensities were measured by densitometric analysis using Image J software. The source and dilution factor of antibodies used were listed in Supplementary Table 1.

### Enzyme-linked immunosorbent assay

Expression levels of mouse TNF-α (Cat#558534; BD Bioscience, Franklin Lakes, NJ), and mouse IL-1β (Cat#DY401; R&D Systems, Minneapolis, MN) were quantified using a commercial ELISA kit. All procedures were carried out following the manufacturer’s instructions.

### Immunofluorescence (IF) staining

Mouse brains were harvested and fixed in PBS-buffered formalin (10%, Sigma-Aldrich) for 24 h. Following fixation, brains were cryoprotected by immersion in a 30% sucrose solution in PBS at 4 °C until the tissue sank, indicating full infiltration. The brains were then embedded in optimal cutting temperature (OCT) compound and rapidly frozen using a dry ice bath. The frozen brain blocks were stored at -80 °C until sectioning. Coronal brain sections of 30 μm were cut on a cryostat at -20 °C. The sections were then mounted onto positively charged glass slides (Thermo-Fisher) and stored at -80 °C. Then, tissue slides were washed with cold PBS and permeabilized using 0.2% Triton X-100 in PBS. Subsequently, tissue slides were blocked with 3% normal goat serum in PBST buffer, then incubated with the antibodies against CD31 (1:200, Abcam, ab182981, RRID: AB_2920881), MPO (1:200, BD Biosciences, 556035, RRID: AB_396309 ), and pan-lysine lactylation (Pan-Kla) (1:500, Huabio, Cat#HA722037, RRID: AB_3096499) at 4 ℃ overnight. Nuclear staining was performed using 4′,6-diamidino-2-phenylindole (DAPI; Cat#C0065; Solarbio, Beijing, China) for 15 min at room temperature. Fluorescent images were captured using a Nikon AXR NSPARC confocal microscope. For immunofluorescence analysis, brains were processed into coronal sections, and representative images were acquired from sections containing the cerebral cortex, corresponding approximately to bregma − 1.7 to − 2.1 mm.

### Statistical analysis

All statistical analyses were performed using GraphPad Prism 9 software. Data are presented as mean ± SD. Student’s t-test was used for comparisons between two groups. For comparisons among multiple groups with one independent variable, one-way ANOVA followed by Bonferroni’s multiple-comparison test was used. For experiments involving two independent variables, such as genotype and treatment, two-way ANOVA followed by an appropriate post hoc multiple-comparison test was applied. A P value < 0.05 was considered statistically significant. Sample sizes were determined based on minimizing animal use while ensuring statistical rigor; cell-based assays used *n* = 4 independent replicates, and animal studies used *n* = 4–6 per group depending on endpoint variability, with no outliers excluded.

## Results

### PTPRO silencing attenuates LPS-induced glycolytic and HIF-1 signaling in brain endothelial cells

To investigate the regulatory role of PTPRO in brain endothelial cells, we first established a PTPRO knockdown model in bEND.3 cells using siRNA targeting PTPRO. Cells were subsequently stimulated with lipopolysaccharide (LPS) for 24 h to recapitulate the inflammatory microenvironment observed in LPS-induced neuroinflammation (Fig. 1A). Western blot analysis confirmed efficient and time-dependent suppression of PTPRO protein expression, with significant reductions observed at 24 and 48 h following siRNA transfection (Fig. 1B–C).

RNA sequencing was then performed in negative control (NC) and PTPRO-silenced (si-PTPRO) bEND.3 cells post LPS challenge. Differential pathway enrichment analysis revealed distinct transcriptional responses between the two groups, comparing LPS-induced effects in NC cells versus those in si-PTPRO cells. The most significantly altered pathways were identified and ranked by differences in p-values. Notably, the majority of top-ranked differentially regulated pathways that were robustly activated by LPS in NC cells were markedly attenuated in si-PTPRO cells (Fig. 1D). These pathways were predominantly associated with cellular metabolism, including glycolysis/gluconeogenesis, HIF-1 signaling, carbon metabolism, and fructose–mannose metabolism (Fig. 1D). In parallel, key inflammatory pathways exhibited a similar pattern of reduced activation in PTPRO-silenced cells, consistent with a dampened inflammatory response (Fig. 1E). In contrast, PTPRO silencing sensitized endothelial responses to LPS in a distinct set of pathways, including homologous recombination, cell cycle regulation, and gastric acid secretion (Supplementary Fig. S1). Collectively, these transcriptomic data demonstrate for the first time that PTPRO silencing significantly attenuates LPS-induced activation of HIF–1α–mediated glycolytic and inflammatory pathways, highlighting a critical role for PTPRO in regulating endothelial metabolic reprogramming and inflammatory signaling under septic conditions.

The top ten metabolism KEGG pathways affected by LPS in NC and si-PTPRO cells were listed for comparison (Fig. 1F–G). Glycolysis/gluconeogenesis signaling were prominently enriched in NC cells but markedly reduced under PTPRO deficiency. Gene Set Enrichment Analysis (GSEA) further confirmed significant enrichment of HIF-1 signaling in control cells (NES = 1.96, FDR = 0.02; Fig. 1H), which was substantially weakened in si-PTPRO cells (NES = 1.42, FDR = 0.22; Fig. 1I) compared to NC cells. Chord diagrams integrating KEGG and GO terms highlighted key genes driving glycolytic and hypoxia-related signaling (Fig. 1J–K). Major glycolytic enzymes, including HK2, PFKP, ALDOC, and LDHA, were coordinately upregulated in both NC cells and si-PTPRO cells upon LPS stimulation, whereas the volume of activated genes in this pathway was reduced by PTPRO knockdown.

Together, these results indicate, for the first time, that PTPRO is essential for full activation of glycolysis and HIF-1α signaling in response to LPS, highlighting a critical role of PTPRO in metabolic reprogramming during LPS-induced inflammatory activation of brain microvascular endothelial cells.

### PTPRO enhances glucose influx and glycolytic activity and increases HIF-1α in LPS-stimulated brain endothelial cells

**Fig. 2:**
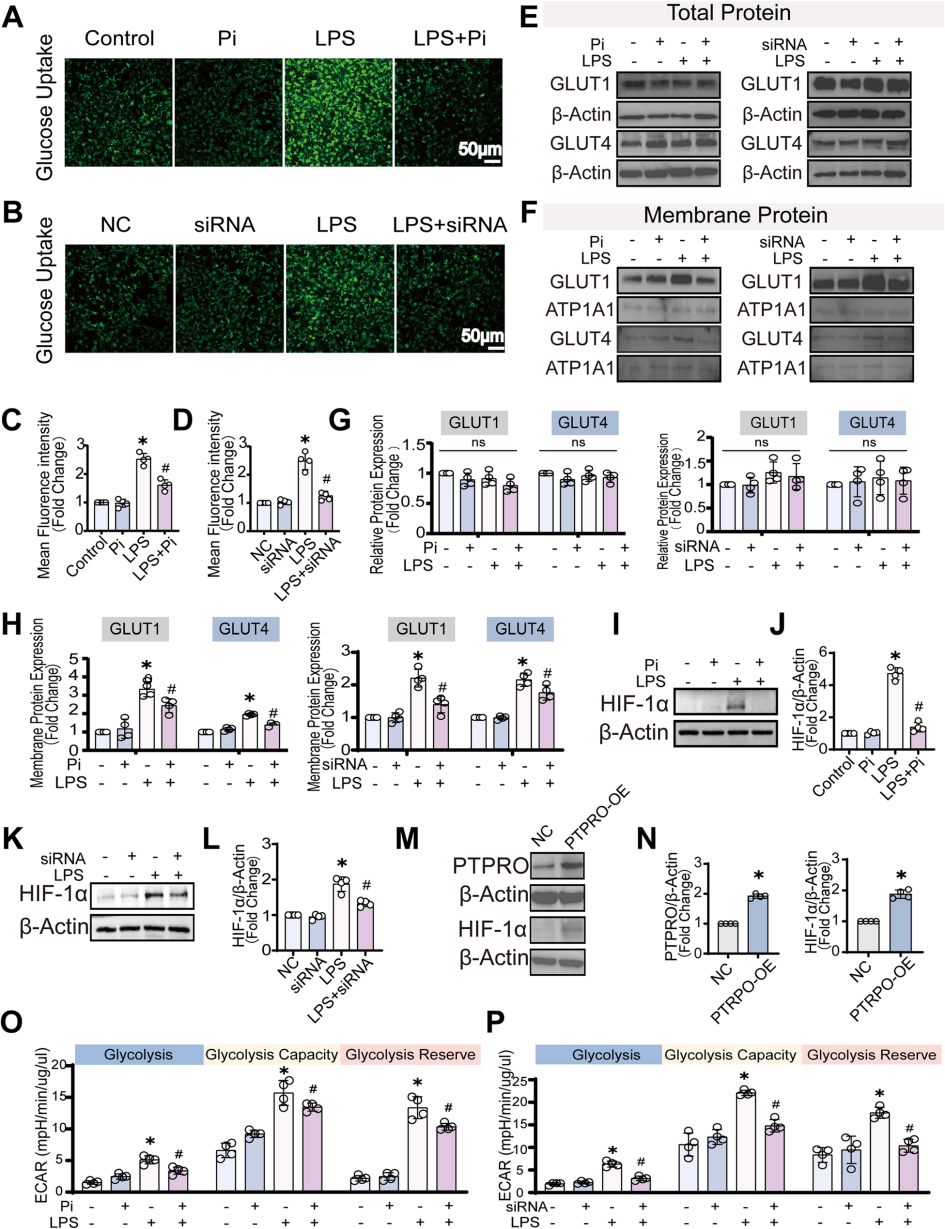
PTPRO enhanced LPS-induced glucose uptake by promoting HIF-1α–dependent GLUT translocation and activation of glycolysis. **A**–**B** Representative immunofluorescence images showing glucose uptake in bEND.3 cells transfected with control or PTPRO siRNAs for 24 h, followed by subsequent stimulation with LPS (500 ng/mL) for an additional 24 h. Pi was added 30 min prior to LPS stimulation as indicated (n = 4). **C**–**D** Quantification of glucose uptake–associated fluorescence intensity from images shown in (A) and (B) (*n* = 4). **E**–**F** Immunoblot analysis of total cellular protein and plasma membrane fractions for GLUT1 and GLUT4 expressions in bEND.3 cells pretreated with Pi (30 min before stimulate) or transfected with PTPRO siRNAs for 24 h, followed by LPS (500 ng/mL) stimulation for 24 h (n = 4). **G**–**H** Densitometric quantification of total and membrane-associated GLUT1 and GLUT4 protein levels corresponding to (E) and (F) (*n* = 4). **I**–**L** Immunoblot analysis and quantification of HIF-1α protein expression in bEND.3 cells pretreated with Pi (30 min) or transfected with PTPRO-siRNA for 24 h, followed by LPS (500 ng/mL) stimulation for 24 h (n = 4). **M**–**N** Immunoblot analysis and quantification of HIF-1α protein expression in bEND.3 cells overexpressing PTPRO (*n* = 4). **O**–**P** Seahorse extracellular acidification rate (ECAR) analysis of glycolysis, glycolytic capacity, and glycolytic reserve in bEND.3 cells stimulated by LPS (500 ng/mL) for 24 h, with Pi pretreatment (30 min) or PTPRO silencing (24 h) as indicated (n = 4). Data are presented as mean ± SD. (* *p* < 0.05 vs. Control groups; ^#^*p* < 0.05 vs. LPS groups; ns, not significant.)

Next the impact of PTPRO on LPS-mediated glycolysis in endothelial cells were validated in cell biology assays. To determine whether the PTPRO-controlled glycolysis during LPS challenge in brain endothelial cells resulted from increased glucose uptake, we measured glucose influx using a fluorescent glucose uptake assay. LPS stimulation significantly elevated intracellular glucose uptake compared with untreated controls, whereas co-treatment with a PTPRO inhibitor (Pi) [16] or PTPRO siRNA partially attenuated this increase (Fig. 2A-D), indicating that PTPRO is required for optimal glucose influx during inflammatory activation of endothelial cells, but not at basal conditions.

Plasma membrane trafficking of glucose transporters glucose transporter-1 (GLUT1) and glucose transporter-4 (GLUT4) are critical for cellular glucose uptake [17]. We next assessed their expression levels and membrane localization (Fig. 2E–F). Total protein levels remained unchanged (Fig. 2G), however, LPS stimulation increased GLUT1 and GLUT4 accumulation at the plasma membrane (Fig. 2H). Analysis of membrane fractions confirmed that LPS promoted translocation of both transporters to the cell surface, consistent with enhanced glucose uptake. In contrast, PTPRO silencing or co-treatment with Pi markedly reduced membrane localization of GLUT1 and GLUT4 compared with LPS-treated controls, indicating that PTPRO is required for proper glucose transporter trafficking during endothelial inflammatory activation (Fig. 2G-H).

HIF-1α, a master transcriptional regulator of glycolysis, mediates metabolic reprogramming from oxidative phosphorylation to glycolysis [18]. We next examined the effect of PTPRO on HIF-1α protein levels to determine whether PTPRO-mediated glycolytic activation during LPS challenge is associated with HIF-1α signaling. LPS robustly increased HIF-1α accumulation, which was attenuated by co-treatment with the PTPRO inhibitor Pi (Fig. 2I-J). Similarly, PTPRO silencing significantly reduced the LPS-induced HIF-1α response (Fig. 2K-L). Consistently, PTPRO overexpression (PTPRO-OE) enhanced HIF-1α levels even in the absence of LPS, with further elevation observed upon LPS stimulation (Fig. 2M-N). Together, these results indicate that PTPRO positively regulates HIF-1α expression, and promotes glycolytic programming in LPS-activated brain endothelial cells.

We next evaluated glycolytic activity by measuring the extracellular acidification rate (ECAR), an indicator of lactate production and glycolytic flux. LPS stimulation enhanced glycolysis, glycolytic capacity, and glycolytic reserve, indicative of a classical shift toward aerobic glycolysis [19]. Treatment with the PTPRO inhibitor markedly reduced all ECAR parameters, reflecting impaired glycolytic engagement (Fig. 2O). Similarly, PTPRO silencing substantially attenuated LPS-induced increases in glycolysis and glycolytic capacity and strongly suppressed glycolytic reserve (Fig. 2P). These findings are consistent with the observed reductions in glucose uptake, glucose transporter membrane localization, and HIF-1α activation upon PTPRO depletion/inhibition.

### Inhibition of PTPRO attenuates glycolytic activation, HIF-1α induction, and neuroinflammatory responses in a murine model of LPS induced neuroinflammation

**Fig. 3:**
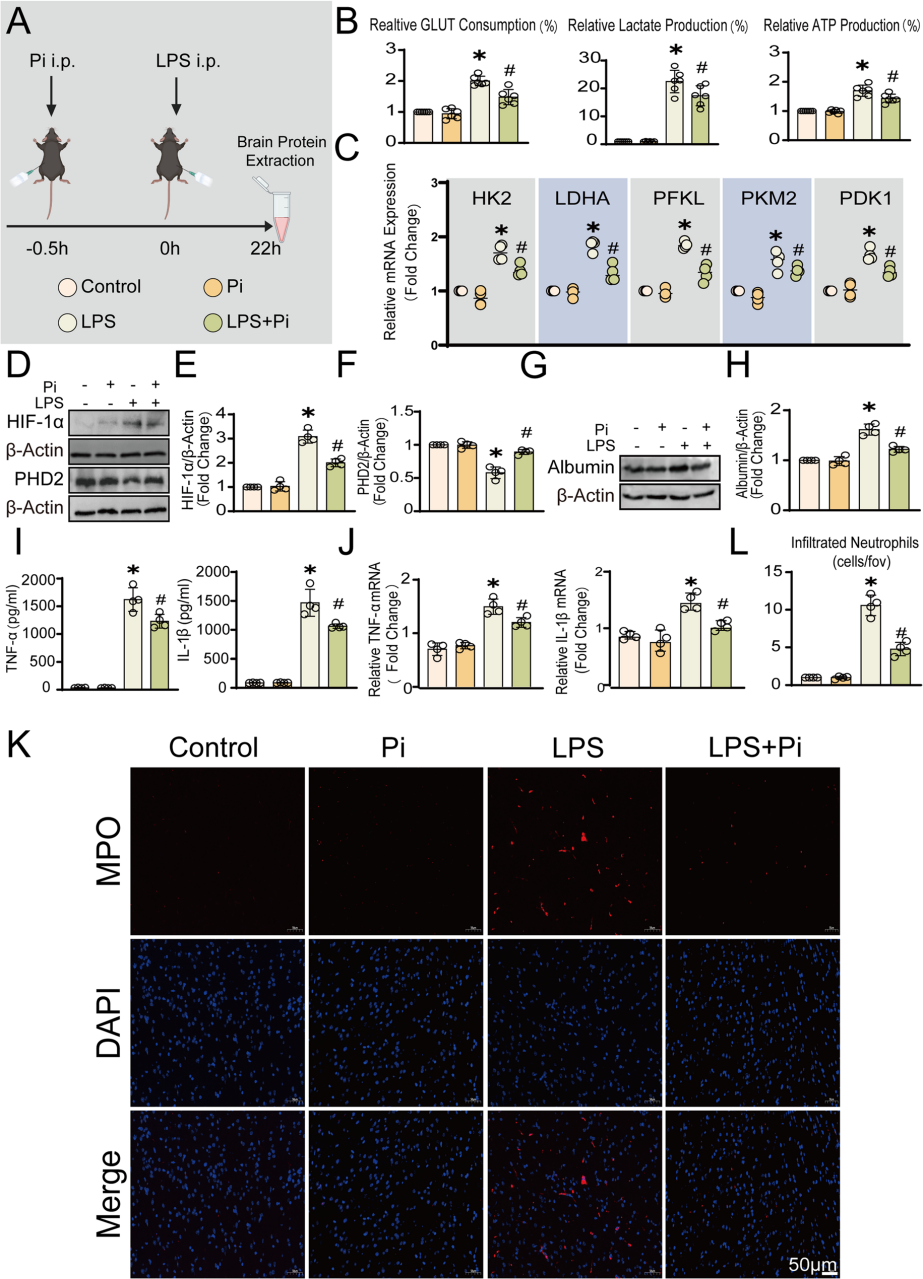
PTPRO modulated LPS-driven metabolic reprogramming, BBB disruption and neuroinflammatory responses. **A** Schematic illustration of the experimental design showing Pi pretreatment followed by LPS injection and subsequent collection of cerebral tissues. Mice received Pi 0.5 h before intraperitoneal (i.p.) injection of LPS (10 mg/kg), and brain tissues were harvested 22 h later. Control mice received an equal volume of PBS. **B** Measurement of glucose consumption, lactate production, and ATP levels in brain tissues from mice subjected to LPS-induced neuroinflammation model (*n* = 6). **C** RT–qPCR analysis of glycolysis-related genes, including HK2, LDHA, PFKL, PKM2, and PDK1, in brain tissues (*n* = 6). **D**–**F** Immunoblot analysis and densitometric quantification of HIF-1α and PHD2 protein levels in brain tissues from mice pretreated with Pi 0.5 h prior to LPS stimulation (10 mg/kg, i.p., 22 h) (n = 4). **G**–**H** Representative immunoblot analysis and quantification of albumin levels in brain tissues (*n* = 4). **I**–**J** ELISA measurement of TNF-α and IL-1β protein levels and RT–qPCR analysis of TNF-α and IL-1β mRNA expressions in mouse brain tissues (*n* = 4). **K**–**L** Representative immunofluorescence images of myeloperoxidase (MPO) staining and quantification of MPO-positive neutrophil infiltration in the cerebral cortex region. Data are presented as mean ± SD. (* *p* < 0.05 vs. Control groups; ^#^*p* < 0.05 vs. LPS groups.)

To further investigate the role of PTPRO in neuroinflammatory dysregulation during sepsis-associated encephalopathy, an LPS-induced neuroinflammation mouse model of SAE was established (Fig. 3A). LPS challenge markedly enhanced glycolysis, as reflected by increased glucose consumption, lactate production, and ATP levels, however, inhibition of PTPRO significantly attenuated these LPS-induced metabolic changes (Fig. 3B). At the transcriptional level, RT-qPCR analysis demonstrated that the expressions of key glycolytic enzymes including HK2, LDHA, PFKL, PKM2, and PDK1) were all significantly upregulated in SAE brain tissue, whereas PTPRO inhibition resulted in a pronounced reduction of these glycolytic genes (Fig. 3C). Given the involvement of HIF-1α in inflammatory glycolytic responses in the central nervous system of SAE, HIF-1α expression was next examined. HIF-1α levels were significantly increased in brain tissues from LPS-induced SAE mice compared with controls. In contrast, inhibition of PTPRO markedly reduced HIF-1α expression following LPS challenge (Fig. 3D-E). In addition, PHD2, the upstream negative regulator of HIF-1α, exhibited an opposite trend compared with HIF-1α levels (Fig. 3F).

Sepsis-associated encephalopathy is characterized by blood brain barrier disruption and enhanced neuroinflammatory responses. We further examined the effect of PTPRO inhibition on BBB integrity and neuroinflammation. PTPRO inhibitor significantly reduced albumin leakage into the brains of SAE mice, indicating that PTPRO inhibition effectively attenuates blood–brain barrier permeability disruption in the SAE model (Fig. 3G-H). Consistently, RT-qPCR and ELISA results showed that PTPRO inhibition markedly decreased the expressions of pro-inflammatory cytokines such as TNF-α and IL-1β in brain tissue (Fig. 3I-J). In addition, PTPRO inhibitor also significantly reduced the number of infiltrating neutrophils in the brain parenchyma of mice. (Fig. 3K-L), further supporting a protective role of PTPRO inhibitor in preserving BBB integrity and limiting neuroinflammatory escalation during SAE.

Collectively, these findings demonstrated that PTPRO promotes metabolic dysregulation, HIF-1α activation, BBB breakdown, and neuroinflammation in SAE, whereas its inhibition confers metabolic and neurovascular protection.

### Systemic PTPRO knockout mitigates glycolytic overactivation and neuroinflammatory responses in SAE

**Fig. 4:**
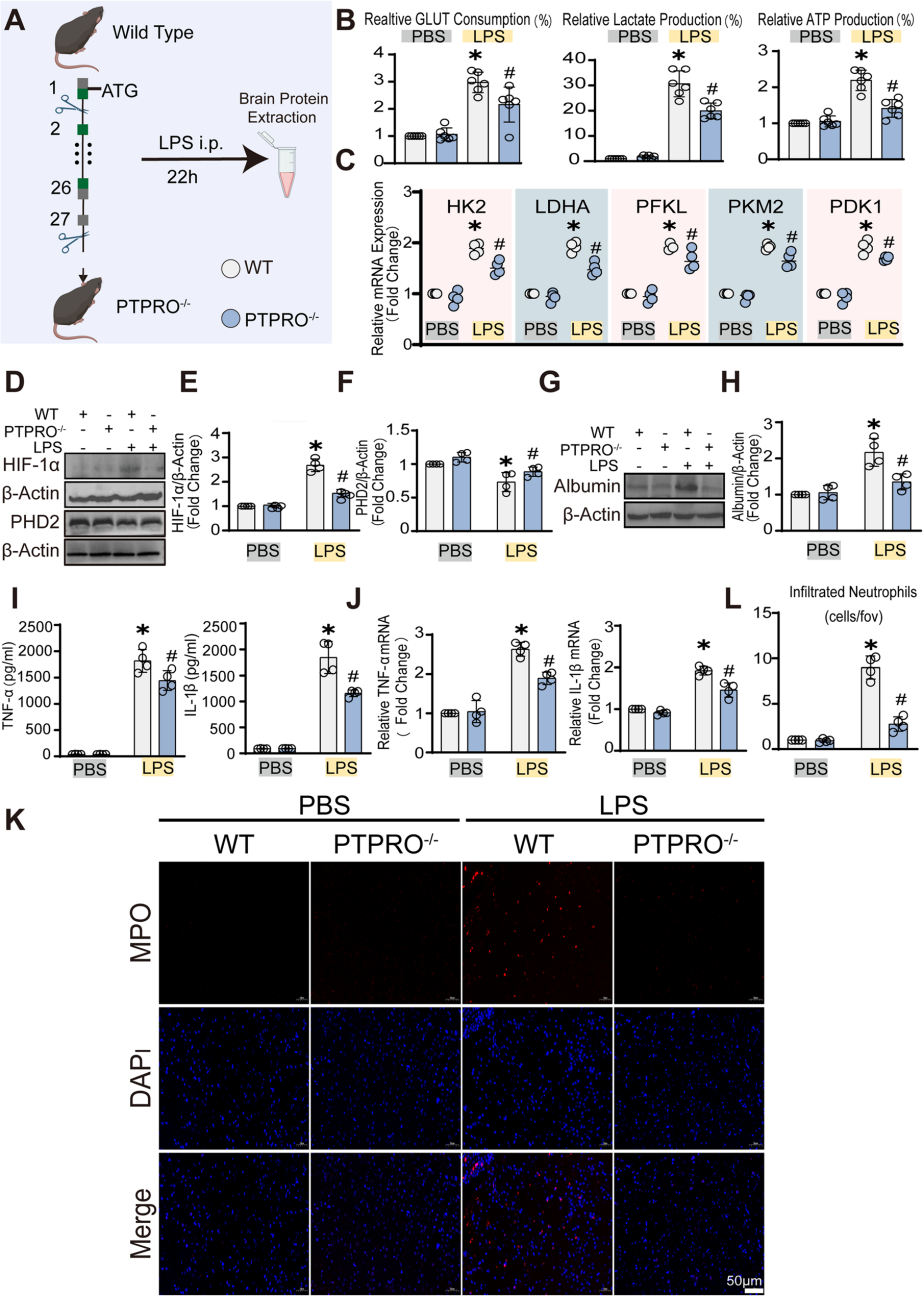
PTPRO deficiency attenuated LPS-induced metabolic reprogramming, BBB disruption, and neuroinflammatory responses in SAE model. **A** Schematic illustration of the experimental design using wild-type (WT) and PTPRO⁻/⁻ mice, followed by intraperitoneal (i.p.) injection of LPS (10 mg/kg) and subsequent brain tissue collection 22 h later. Control mice received an equal volume of PBS. **B** Measurement of glucose consumption, lactate production, and ATP levels in brain tissues from mice after LPS administration (*n* = 6). **C** RT–qPCR analysis of glycolysis-related genes, including HK2, LDHA, PFKL, PKM2, and PDK1, in brain tissues from WT and PTPRO⁻/⁻ mice (*n* = 6). **D**–**F** Immunoblot analysis and densitometric quantification of HIF-1α and PHD2 protein levels in brain tissues under LPS stimulation (10 mg/kg, i.p., 22 h) (n = 4). **G**–**H** Representative immunoblot analysis and quantification of albumin levels in brain tissues (*n* = 4). **I**–**J** ELISA measurement of TNF-α and IL-1β protein levels and RT–qPCR analysis of TNF-α and IL-1β mRNA expressions in mouse brain tissues (*n* = 4). **K**–**L** Representative immunofluorescence images of myeloperoxidase (MPO) staining and quantification of MPO-positive neutrophil infiltration in the cerebral cortex region. Data are presented as mean ± SD. (*p < 0.05 vs. Control groups; ^#^*p* < 0.05 vs. LPS groups.)

To further determine the contribution of PTPRO to metabolic and inflammatory burden during SAE and rule out the possible off-target effects of Pi, PTPRO^−/−^ mice were subjected to LPS-induced SAE (Fig. 4A). We first assessed the effects of PTPRO knockout on metabolic reprogramming within the brain. As expected, LPS administration significantly increased glucose consumption, lactate production, and ATP turnover in the brains of WT mice, indicative of enhanced glycolytic flux. In contrast, these metabolic changes were significantly attenuated in PTPRO^−/−^ mice following LPS challenge (Fig. 4B). Consistently, RT-qPCR analysis demonstrated a marked upregulation of glycolysis-related genes (HK2, LDHA, PFKL, PKM2, and PDK1) in the brains of WT mice with SAE, whereas their expressions were significantly reduced in PTPRO^−/−^ mice (Fig. 4C).

Then HIF-1α expression was subsequently examined in glycolytic activation. WT mice exhibited a pronounced increase in HIF-1α following LPS stimulation, whereas this response was markedly curtailed in PTPRO^−/−^ animals (Fig. 4D-E). Correspondingly, PHD2 displayed an inverse regulatory pattern, further reinforcing the notion that PTPRO influences HIF-1α stabilization during LPS induced neuroinflammation (Fig. 4F). We then evaluated whether global PTPRO deficiency also preserved blood–brain barrier function, a key determinant of neuroinflammation severity in SAE. PTPRO^−/−^ mice showed markedly decreased albumin extravasation and attenuated vascular hyperpermeability under septic conditions (Fig. 4G-H). In parallel, quantitative analyses demonstrated that deletion of PTPRO significantly lowered the production of central pro-inflammatory cytokines TNF-α and IL-1β (Fig. 4I-J). Moreover, neutrophil recruitment into the cerebral cortex region, a hallmark of neuroinflammation, was significantly reduced in PTPRO^−/−^ mice (Fig. 4K-L). Of note, these findings revealed that systemic ablation of PTPRO alleviates LPS-induced metabolic stress, restrains HIF-1α-driven inflammatory activation, preserves BBB integrity, and limits neuroinflammatory progression. These observations are consistent with the findings from PTPRO inhibitor described above, and support a broader role for PTPRO in LPS induced neuroinflammation.

### Brain endothelial PTPRO drives glycolytic reprogramming and neuroinflammation in LPS induced neuroinflammation

**Fig. 5:**
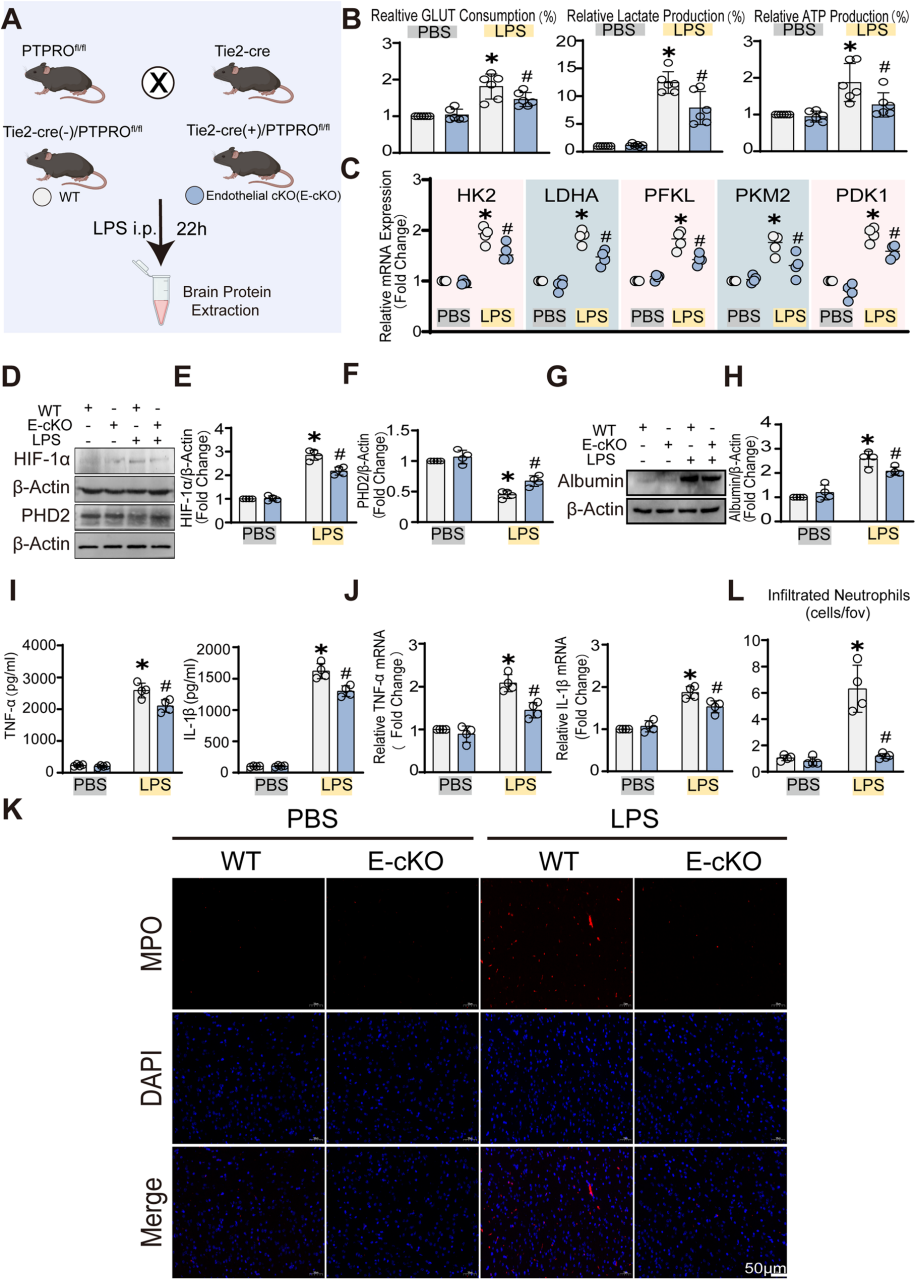
Endothelial-specific deletion of PTPRO mitigated LPS-induced metabolic reprogramming, BBB disruption, and neuroinflammation. **A** Schematic illustration of the experimental design using WT and E-cKO mice. Mice received intraperitoneal (i.p.) injection of LPS (10 mg/kg), and brain tissues were collected 22 h later. Control mice received an equal volume of PBS. **B** Measurement of glucose consumption, lactate production, and ATP levels in brain tissues (*n* = 6). **C** RT–qPCR analysis of glycolysis-related genes, including HK2, LDHA, PFKL, PKM2, and PDK1, in brain tissues from WT and E-cKO mice (*n* = 6). **D**–**F** Immunoblot analysis and densitometric quantification of HIF-1α and PHD2 protein levels in brain tissues under LPS stimulation (10 mg/kg, i.p., 22 h) (n = 4). (**G**–**H**) Representative immunoblot analysis and quantification of albumin levels in brain tissues (*n* = 4). (**I**–**J**) ELISA measurement of TNF-α and IL-1β protein levels and RT–qPCR analysis of TNF-α and IL-1β mRNA expression in brain tissues (*n* = 4). (**K**–**L**) Representative immunofluorescence images of myeloperoxidase (MPO) staining and quantification of MPO-positive neutrophil infiltration in the cerebral cortex region (*n* = 4). Data are presented as mean ± SD. (*p < 0.05 vs. Control groups; ^#^*p* < 0.05 vs. LPS groups.)

It is well documented that glycolytic reprogramming and permeability alterations in endothelial cells are critical to the progression of SAE [20]. To confirm that the phenotype above was mediated by brain endothelial PTPRO during SAE, we first generated endothelial-specific PTPRO knockout mice (E-cKO mice) (Fig. 5A). Consistent with previous observations, LPS significantly increased glycolytic activity, as evidenced by elevated glucose consumption, lactate production, and altered ATP levels, and these metabolic changes were markedly attenuated in E-cKO mice (Fig. 5B). Consistently, RT-qPCR analysis revealed that the expressions of key glycolytic enzymes were upregulated in SAE brains from WT mice but significantly reduced in E-cKO mice (Fig. 5C). Consistent with the metabolic changes observed above, HIF-1α expression was markedly elevated in LPS-induced SE mice, which was substantially attenuated in E-cKO mice (Fig. 5D-E). The opposite trend observed for PHD2 further supported the regulatory role of endothelial PTPRO in modulating HIF-1α stability during SAE, thereby influencing SAE progression (Fig. 5F). We next assessed the effects of endothelial PTPRO deletion on blood–brain barrier permeability. E-cKO mice exhibited significantly reduced albumin leakage and alleviated vascular hyperpermeability in the SAE model (Fig. 5G-H). In parallel, RT-qPCR and ELISA analyses showed that endothelial knockout of PTPRO effectively suppressed the expression of pro-inflammatory cytokines TNF-α and IL-1β in the brain tissue (Fig. 5I-J). Furthermore, Neutrophil infiltration into the central nervous system was markedly reduced in E-cKO mice (Fig. 5K-L), providing additional evidence that endothelial PTPRO deletion preserves BBB integrity and restricts neuroinflammatory progression in SAE. These results indicate that endothelial PTPRO drives metabolic reprogramming and exacerbates neuroinflammation in septic encephalopathy, while endothelial PTPRO knockout provides metabolic, neuroprotective, and anti-inflammatory benefits. To assess whether neutrophil-specific deletion of PTPRO affects blood-brain barrier permeability in response to LPS, we measured albumin leakage into the brain parenchyma and TNF-α levels 22 h after intraperitoneal LPS injection. The albumin content in the brain parenchyma was comparable between neutrophil-specific PTPRO conditional knockout mice and WT mice, with no statistically significant difference detected (Supplementary Fig. S2). These findings indicate that neutrophil-specific deletion of PTPRO does not significantly alter blood-brain barrier permeability under LPS-induced inflammatory conditions.

### Endothelial PTPRO regulates ErbB2 phosphorylation and inflammatory AKT-mTOR activation to modulate glycolytic reprogramming and BBB dysfunction

**Fig. 6:**
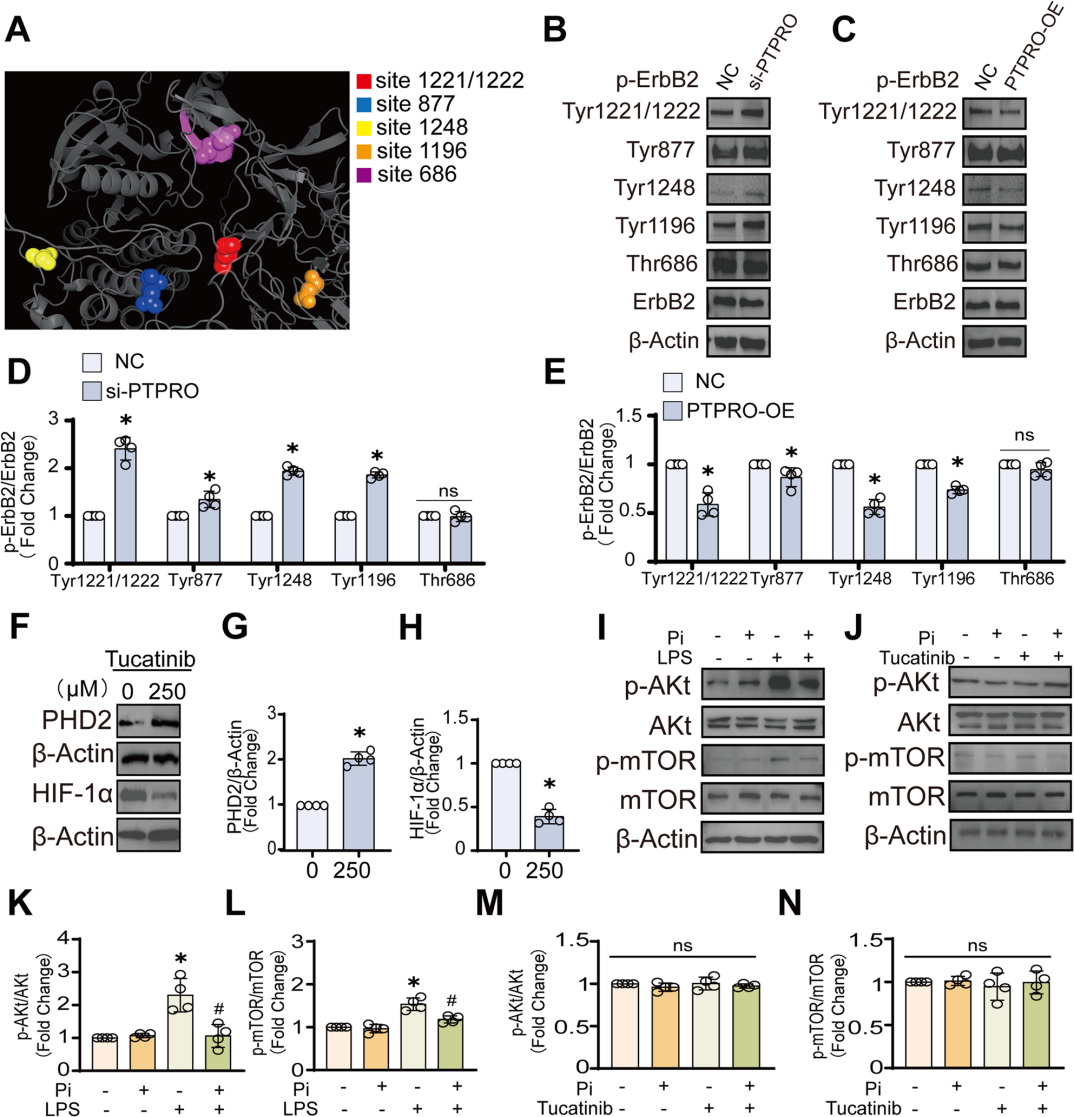
PTPRO modulated LPS-induced metabolic reprogramming and neuroinflammation through the ErbB2-Akt-mTOR-HIF-1α axis. **A** Structural depiction of ErbB2 domains generated by PyMOL. **B**–**E** Immunoblot analysis of ErbB2 phosphorylation at the indicated residues in bEND.3 cells following PTPRO silencing (24 h) or stable overexpression via lentiviral transduction (*n* = 4). **F**–**H** Immunoblot analysis and quantification of HIF-1α and PHD2 protein levels in cells treated with the ErbB2 inhibitor tucatinib (250 μM, 30 min) (*n* = 4). **I**–**N** For LPS stimulation experiments (**I**), cells were exposed to LPS (500 ng/mL) for 4 h. Pi (20 mM) was added 30 min before LPS stimulation. In sequential treatment experiments (**J**), cells were first treated with tucatinib for 30 min followed by Pi for an additional 30 min. Immunoblot analysis of components of the Akt–mTOR signaling pathway in cells subjected to PTPRO modulation and/or tucatinib treatment as indicated (*n* = 4). Data are presented as mean ± SD. (*P < 0.05 vs. NC; #*P* < 0.05 vs. LPS; ns, no significance.)

To elucidate how PTPRO regulates endothelial metabolism and neuroinflammation, we examined its downstream signaling. As PTPRO negatively regulates ErbB2 by dephosphorylating it [12], thereby modulating downstream signaling pathways such as PI3K/AKT and MAPK/ERK to control endothelial metabolism and inflammatory responses. Although it is known PTPRO can dephosphorylate ErbB2, the specific tyrosine residue of ErbB2 that is selectively dephosphorylated by PTPRO in brain endothelial cells is unknown. We selected five well-reported phosphorylation sites of ErbB2 (Tyr1221/1222, Tyr 877, Tyr1248, Tyr1196, and Thr686), structural mapping of ErbB2 revealed that the Thr686 residue is spatially separated from the other four phosphorylation sites (Fig. 6A). Moreover, as Thr686 represents a serine/threonine phosphorylation site rather than a tyrosine residue, it is less likely to be directly involved in PTPRO-dependent regulation of ErbB2 tyrosine phosphorylation.

Importantly, PTPRO regulation of ErbB2 phosphorylation extends beyond the previously reported Tyr1248 site, with Tyr1196, Tyr1221/1222, and Tyr877 showing pronounced and reproducible changes upon PTPRO knockdown or overexpression. (Fig. 6B-E). These observations indicate that PTPRO modulates multiple tyrosine residues on ErbB2 in endothelial cells, thereby broadening the current understanding of the PTPRO-ErbB2 signaling axis in the context of neurovascular regulation.

To investigate whether ErbB2 activity mediates the glycolytic response downstream of PTPRO, we treated cells with the ErbB2 inhibitor tucatinib. Similar to PTPRO inhibition, tucatinib (250 μM) effectively suppressed HIF-1α accumulation, while concurrently upregulating PHD2 expression (Fig. 6F–H). The AKT-mTOR pathway, downstream of ErbB2, and also known as a key immune–metabolic axis linking inflammatory signals to glycolytic reprogramming [21], amplifies neuroinflammation and BBB dysfunction under pathological conditions. We next examined whether PTPRO inhibition modulates AKT–mTOR signaling following LPS stimulation. LPS markedly increased AKT and mTOR phosphorylation, whereas PTPRO inhibition significantly attenuated this activation without affecting total AKT or mTOR levels, indicating a role for PTPRO in AKT–mTOR signaling under inflammatory conditions (Fig. 6I, K–L). We further assessed the involvement of ErbB2 in this process. Under basal conditions, dual inhibition did not significantly alter AKT or mTOR phosphorylation. In contrast, suppression of AKT-mTOR signaling was observed only in LPS-stimulated cells (Fig. 6J, M-N). These data suggest that ErbB2 is downstream of PTPRO in Akt-mTOR signaling regulation upon LPS challenge. Together, these findings demonstrated that PTPRO-ErbB2-mediated regulation of HIF-1α mediated glucose metabolism signaling is contingent on inflammatory activation rather than basal ErbB2 expression, supporting a dependent role of this pathway in metabolic reprogramming.

### PTPRO couples glycolytic reprogramming to endothelial inflammation via HIF-1α

**Fig. 7:**
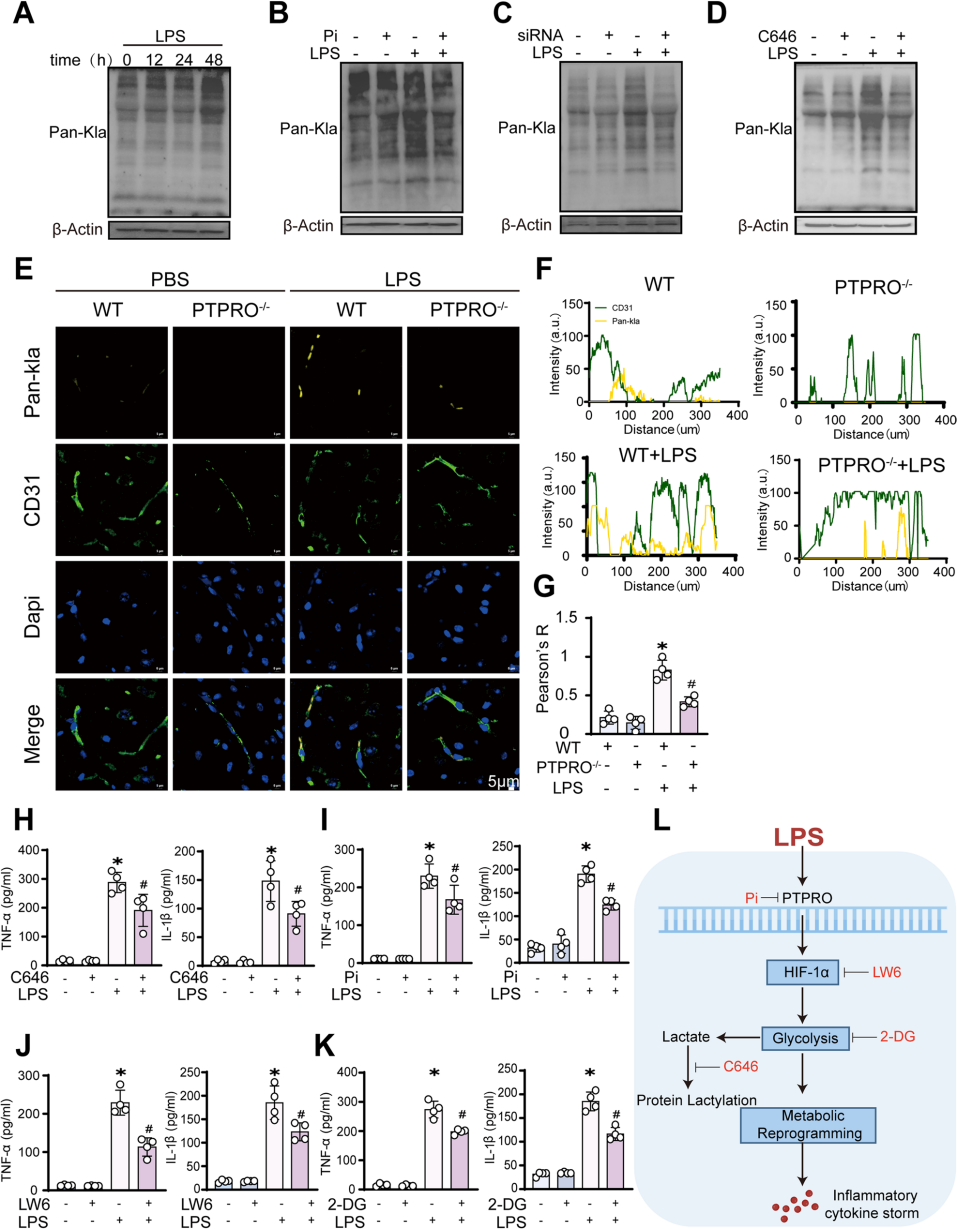
PTPRO inhibition restrained LPS-induced inflammation by limiting endothelial glycolytic flux and lactate production. **A** Immunoblot analysis of pan-lysine lactylation (Pan-Kla) in bEND.3 cells collected at the indicated time points following LPS stimulation (500 ng/mL). **B**–**C** Immunoblot analysis of Pan-Kla levels in cells pretreated with Pi (30 min) or transfected with PTPRO siRNAs prior to LPS stimulation (500 ng/mL, 24 h) (*n* = 4). **D** Immunoblot analysis of Pan-Kla levels in cells treated with the acetyltransferase inhibitor C646 (10 µM, 24 h) followed by LPS stimulation (500 ng/mL, 24 h) (*n* = 4). **E**–**G** Representative immunofluorescence staining and quantitative analysis of CD31 and Pan-Kla in brain tissues. Pan-Kla staining reflects lactate accumulation and its spatial distribution in the brain following LPS challenge (*n* = 4). **H**–**K** Measurement of TNF-α and IL-1β levels by ELISA in cells pretreated with the p300/CBP histone acetyltransferase inhibitor C646 (10 µM, 24 h), PTPRO inhibitor Pi (20mM, prior 30 min then LPS stimulate), the HIF-1α inhibitor LW6 (10 µM, 24 h), or the glycolytic inhibitor 2-deoxy-D-glucose (2-DG, 5 mM, 24 h), followed by LPS stimulation (500 ng/mL, 24 h) (*n* = 4). **L** Schematic diagram illustrating the proposed anti-inflammatory and neuroprotective mechanisms of PTPRO inhibition through regulation of glycolysis, protein lactylation, and metabolic reprogramming in SAE. Data are presented as mean ± SD. (*P < 0.05 vs. control; #*P* < 0.05 vs. LPS.)

Excessive lactate accumulation resulting from sustained glycolytic activation promotes protein lactylation, a relatively long-lasting post-translational modification that contributes to persistent endothelial inflammation during sepsis [22]. To establish a mechanistic link between PTPRO-driven glycolysis and endothelial inflammatory activation, we next examined protein lactylation as a downstream consequence of enhanced glycolytic flux. LPS stimulation induced a time-dependent increase in global protein lactylation in brain endothelial cells, with maximal levels observed at 48 h (Fig. 7A). Notably, both pharmacological inhibition and genetic silencing of PTPRO significantly reduced LPS-induced lactate production (Fig. 7B–C). In addition, inhibition of the p300/CBP histone lactyl-transferase using C646 [23] markedly suppressed lactate accumulation under LPS stimulation (Fig. 7D). Immunofluorescence analysis further demonstrated increased lactate accumulation in brain endothelial cells within the cerebral parenchyma of LPS-challenged mice. In contrast, endothelial lactate signals were substantially diminished in PTPRO-deficient mice following LPS exposure, indicating that PTPRO is required for inflammation-induced lactate production in vivo (Fig. 7E–G). Together, these data support a role for PTPRO in promoting LPS induced persistent endothelial inflammation through enhanced glycolysis and lactate-dependent signaling.

To further determine whether PTPRO-driven metabolic reprogramming is functionally required for sustained septic inflammation, we next evaluated the effects of key pathway inhibitors on cytokine production during LPS-induced inflammatory activation. Endothelial cells were treated with a protein lactylation inhibitor (C646), a HIF-1α inhibitor (LW-6), a glycolytic inhibitor (2-deoxy-D-glucose, 2-DG), or a PTPRO inhibitor prior to LPS stimulation, followed by measurement of pro-inflammatory cytokines, including TNF-α and IL-1β. Inhibition of protein lactylation with C646 markedly suppressed LPS-induced cytokine expression (Fig. 7H). Pharmacological inhibition of PTPRO significantly reduced TNF-α and IL-1β levels (Fig. 7I). Similarly, HIF-1α inhibition robustly attenuated pro-inflammatory cytokine production, indicating a requirement for HIF-1α activity in sustaining endothelial inflammatory signaling (Fig. 7J). Consistent with these findings, blockade of glycolysis with 2-DG also significantly reduced cytokine expression, supporting a critical role for glycolytic reprogramming in endothelial inflammatory responses (Fig. 7K). To further examine whether PTPRO, HIF-1α, glycolysis, and lactylation act through a shared inflammatory pathway rather than through parallel independent mechanisms, we performed double-inhibition experiments in LPS-stimulated bEND.3 cells using Pi combined with LW6, C646, or 2-DG. Notably, combined inhibition did not produce a statistically significant additional reduction in TNF-α and IL-1β secretion compared with the corresponding single-inhibitor treatments (Supplementary Figure S3). These non-additive effects support a model in which PTPRO, HIF-1α, glycolytic activity, and lactylation are functionally linked within the same inflammatory signaling axis in LPS-stimulated brain endothelial cells.

In summary, these findings demonstrate that PTPRO promotes endothelial inflammatory activation through a HIF-1α–dependent glycolytic and lactate-driven pathway. Inhibition of PTPRO, HIF-1α, or glycolysis reduced LPS-induced lactate accumulation and suppressed pro-inflammatory cytokine production. Concordant in vitro and in vivo evidence identifies PTPRO as a central regulator coupling metabolic reprogramming to persistent endothelial inflammation during sepsis. A schematic model illustrating the proposed signaling cascade linking PTPRO activity to glycolytic reprogramming, protein lactylation, and downstream inflammatory responses is presented in Fig. 7L.

## Discussion

In this study, we demonstrate that PTPRO plays a pivotal role in driving LPS-induced metabolic reprogramming and neuroinflammation in sepsis-associated encephalopathy, primarily through its actions in brain endothelial cells. Using a combination of in vitro models with bEND.3 cells and in vivo LPS-induced SAE models in wild-type, systemic PTPRO knockout, and endothelial-specific PTPRO conditional knockout mice, we found that PTPRO promotes glycolytic flux, as evidenced by enhanced glucose uptake, GLUT1/4 translocation, lactate production, and upregulation of glycolytic enzymes (e.g., HK2, LDHA, PFKL, PKM2, PDK1), while also stabilizing hypoxia-inducible factor-1α (HIF-1α) and suppressing its negative regulator PHD2. Mechanistically, PTPRO modulates multiple tyrosine phosphorylation sites on ErbB2, activating downstream AKT-mTOR signaling under inflammatory conditions, which in turn couples metabolic shifts to pro-inflammatory cytokine production (TNF-α, IL-1β), blood-brain barrier disruption, and neutrophil infiltration. Key innovations include the first identification of PTPRO as an endothelial metabolic checkpoint in SAE, the elucidation of its multi-site regulation of ErbB2 beyond previously reported residues, and the demonstration of lactate-mediated amplification of inflammation via epigenetic lactylation. These findings underscore PTPRO’s significance as a novel therapeutic target, offering potential strategies to mitigate SAE’s neurovascular and inflammatory pathologies by interrupting this integrated signaling axis, addressing a critical gap in sepsis-related brain injury management where current treatments remain largely supportive.

These findings should be considered in the context of a substantial body of work demonstrating that SAE is fundamentally a disorder of neurovascular dysfunction [2], in which early alterations in cerebral microcirculation contribute to downstream neuronal injury. Previous studies have shown that endotoxemia induces impaired neurovascular coupling, reduced capillary perfusion, and endothelial activation, all of which precede overt neuronal dysfunction [24]. In clinically relevant models, sepsis has been associated with decreased functional capillary density and impaired cerebral blood flow regulation, highlighting the vulnerability of the cerebral microvasculature to systemic inflammatory stress [25]. These microvascular disturbances are further linked to tissue hypoxia and metabolic dysregulation in the brain, particularly under conditions of hemodynamic instability [26]. More recent work has also emphasized that endothelial inflammatory signaling pathways actively shape vascular injury, with regulators such as SOCS3 modulating leukocyte adhesion and endothelial activation during endotoxemia [27]. Together, these studies establish that the brain endothelium is not merely a passive barrier but a central driver of SAE pathophysiology.

In parallel, advances in transcriptomic profiling have revealed that endothelial cells undergo dynamic and context-dependent transcriptional reprogramming in response to inflammatory stimuli. Cleuren et al. demonstrated that endothelial responses are highly heterogeneous across vascular beds and often masked in bulk tissue analyses, underscoring the importance of cell-type–specific investigation [28]. Consistent with this, Allen et al. showed that non-neuronal cells, including endothelial populations, exhibit robust and spatially organized transcriptional responses during neuroinflammation [29]. More recently, mechanistic studies have further highlighted that endothelial signaling pathways directly govern inflammatory outcomes; for example, endothelial STING/STAT1 signaling has been shown to drive IL-6–dependent transcriptional programs during endotoxemic shock [30]. These findings collectively support a paradigm in which endothelial cells actively orchestrate neuroinflammation through coordinated transcriptional and signaling networks.

A central innovation in our study lies in the unbiased identification of PTPRO’s link to glucose metabolism dysregulation through comprehensive transcriptomic analysis. RNA sequencing of LPS-challenged bEND.3 brain endothelial cells revealed that PTPRO silencing profoundly reshaped the transcriptional landscape, with the top modulated pathways, including glycolysis/gluconeogenesis, HIF-1 signaling, carbon metabolism, and fructose-mannose metabolism, being predominantly suppressed compared to controls, where these pathways were robustly activated. This data-driven approach, supported by KEGG enrichment, GSEA, and chord diagrams highlighting key glycolytic enzymes (e.g., HK2, PFKP, ALDOC, LDHA) and HIF-1α targets, establishes PTPRO as a critical upstream regulator of endothelial metabolic reprogramming under septic stress. The HIF-1α-regulated metabolic switch toward aerobic glycolysis emerges as pivotal for inflammatory outcomes for several reasons: first, it sustains energy demands during inflammation by upregulating glucose uptake and glycolytic flux; second, it promotes lactate accumulation, which amplifies pro-inflammatory signaling through mechanisms such as histone lactylation and epigenetic modulation; third, it directly couples metabolic adaptations to endothelial activation, leading to enhanced cytokine production (e.g., TNF-α, IL-1β), BBB permeability, and leukocyte infiltration, thereby exacerbating neuroinflammation and SAE progression.

This study provided some convincing evidence positioning ErbB2 (also known as HER2) as a major downstream effector of PTPRO in brain endothelial cells during SAE. Prior studies have established that PTPRO directly dephosphorylates ErbB2, particularly at tyrosine residue Y1248, leading to suppression of ErbB2 signaling, endosomal internalization, and degradation in other cellular contexts such as breast cancer. Our work extends this interaction by demonstrating, for the first time in endothelial cells under inflammatory stress, that PTPRO modulates multiple tyrosine phosphorylation sites on ErbB2, including Tyr1221/1222, Tyr877, Tyr1248, and Tyr1196, beyond the single site previously reported. These phosphorylation changes may be direct, as previously reported, or indirect; this distinction will require further investigation. This broad regulatory impact was confirmed through targeted immunoblotting following PTPRO silencing or overexpression, revealing pronounced and reproducible changes in phosphorylation status. Pharmacological inhibition of ErbB2 with tucatinib recapitulated the effects of PTPRO suppression by reducing HIF-1α accumulation, upregulating PHD2, and attenuating AKT-mTOR activation specifically under LPS-stimulated conditions, indicating that ErbB2 functions downstream of PTPRO in an inflammation-dependent manner. ErbB2 is critical in this SAE setting because its aberrant activation in brain endothelial cells has been implicated in sepsis-induced vascular dysfunction: elevated ErbB2 signaling promotes endothelial-to-mesenchymal transition, increases vascular permeability, and drives pro-inflammatory cytokine release, all of which contribute to BBB disruption and neuroinflammation in SAE. By bridging PTPRO to this well-characterized immune-metabolic axis, ErbB2 emerges as an essential mediator that amplifies the pathological cascade in SAE, linking receptor tyrosine kinase signaling to metabolic reprogramming, HIF-1α stabilization, enhanced glycolytic flux, and sustained endothelial activation, thereby reinforcing the therapeutic rationale for targeting upstream PTPRO-ErbB2 interactions to interrupt endothelial inflammation and neurovascular injury in sepsis-related brain injury.

To further substantiate the endothelial cell-autonomous role of PTPRO in LPS induced neuroinflammation pathogenesis, we employed endothelial-specific PTPRO conditional knockout (E-cKO) mice generated via Tek-Cre-mediated recombination, which selectively ablates PTPRO in vascular endothelial cells while preserving its expression in other tissues. Although Tie2-Cre can also recombine in hematopoietic cells and might lead to reduction of PTPRO in leukocytes [31–33], while global PTPRO deficiency did not reduce circulating neutrophil abundance [34], arguing at least neutrophil-intrinsic PTPRO loss does not cause neutrophil depletion. To directly test whether the observed phenotype could be explained by neutrophil-lineage PTPRO deletion, we generated Elane-Cre–mediated PTPRO conditional knockout mice (N-cKO), a well-established model in which Cre recombinase is expressed under control of the endogenous neutrophil elastase (Elane) locus, enabling gene deletion in neutrophil-lineage/myeloid precursor cells (MGI:2182177). These mice did not show protection against LPS-induced BBB disruption as assessed by brain albumin leakage and TNF-α production (Supplementary Figure S2). These findings, combined with protective effects observed in systemic PTPRO knockout and pharmacological inhibition, suggest a selective role of endothelial PTPRO to facilitate the outcome in a LPS-challenged condition, including glycolytic reprogramming (reduced glucose consumption, lactate production, and glycolytic gene expression), HIF-1α stabilization, BBB integrity (decreased albumin leakage), pro-inflammatory cytokine levels (TNF-α, IL-1β), and neutrophil infiltration into the brain parenchyma following LPS challenge. By isolating the contribution of endothelial PTPRO, these findings demonstrate that brain microvascular endothelial cells serve as the primary mediators of PTPRO-driven metabolic and inflammatory dysregulation in SAE, rather than off-target effects from immune cells or neurons, while not excluding additional contributions from hematopoietic cells. Extending beyond PTPRO, this underscores the broader centrality of endothelial inflammation in SAE etiology: cerebral endothelial cells, as the frontline interface between systemic sepsis and the CNS, undergo inflammatory activation that not only disrupts BBB tight junctions and promotes leukocyte transmigration but also amplifies neuroinflammatory cascades through cytokine release and metabolic byproducts like lactate, which can perpetuate glial activation and neuronal injury. While PTPRO represents a novel upstream integrator in this process, emerging evidence implicates other endothelial regulators—such as TLR4 signaling, NF-κB pathways, and additional receptor tyrosine phosphatases-in similar metabolic-inflammatory crosstalk, suggesting that therapeutic strategies targeting endothelial homeostasis could broadly mitigate SAE’s multifaceted brain injury, addressing a critical unmet need in sepsis management.

One major observed weakness of the study is the use of an immortalized brain endothelial cell line rather than primary endothelial cells. While primary cells offer higher physiological relevance, we selected the bEND.3 cell line due to its stability, reproducibility, and suitability for genetic manipulation, which are essential for mechanistic studies of PTPRO signaling. Importantly, immortalized brain endothelial models such as bEND.3 have been widely used in blood–brain barrier research and have been shown to retain key endothelial phenotypes and barrier characteristics comparable to primary cells [35], while enabling consistent experimental manipulation. Moreover, the central findings from our in vitro experiments were consistently validated in vivo using systemic and endothelial-specific PTPRO knockout models, supporting their physiological relevance. Nonetheless, future studies using primary brain endothelial cells will be important to further strengthen the translational relevance of these findings.

A pivotal advancement in our study is the validation of pharmacological PTPRO inhibition as a valid and potentially novel therapeutic approach for SAE. We employed a specific PTPRO inhibitor (Pi) [16], administered intraperitoneally 30 min prior to LPS challenge, which closely mirrored the protective phenotypes observed in both systemic PTPRO knockout and endothelial-specific conditional knockout mice. This included significant attenuation of LPS-induced glycolytic overactivation (reduced glucose consumption, lactate production, ATP turnover, and glycolytic enzyme expression), decreased HIF-1α stabilization with reciprocal upregulation of PHD2, preservation of BBB integrity (diminished albumin extravasation), suppression of pro-inflammatory cytokine production (TNF-α and IL-1β at both mRNA and protein levels), and reduced neutrophil infiltration into the brain parenchyma. These consistent multi-level benefits across genetic and pharmacological models rule out off-target effects and confirm on-target specificity, while the inhibitor’s pre-treatment efficacy highlights its potential for rapid intervention in acute septic settings. Notably, PTPRO inhibitors have shown promise in related inflammatory contexts, such as sepsis-induced acute lung injury and obesity-associated hepatic steatosis, where compounds like Pi or AKB9775 (selective PTPRZ1 inhibitor) [36] have mitigated inflammation and tissue damage. In the context of SAE, where no targeted therapies currently exist beyond supportive care, PTPRO inhibition emerges as a novel strategy that simultaneously addresses endothelial metabolic reprogramming, neurovascular protection, and neuroinflammation. This offers a disease-modifying mechanism that could improve acute outcomes and potentially reduce long-term cognitive sequelae in sepsis survivors. Future studies optimizing inhibitor pharmacokinetics, dosing regimens, and combination therapies will be essential to translate this endothelial-centric approach into clinical benefit.

In conclusion, this study establishes PTPRO as a novel endothelial metabolic checkpoint that orchestrates LPS-induced glycolytic reprogramming, HIF-1α stabilization, and neuroinflammatory escalation in SAE through the ErbB2–AKT–mTOR axis. By integrating unbiased transcriptomics, multi-model genetic knockouts (systemic and endothelial-specific), and pharmacological inhibition, we demonstrate that PTPRO drives BBB disruption, cytokine production, and neutrophil infiltration, while its suppression confers robust metabolic, neurovascular, and anti-inflammatory protection. These findings highlight the central role of brain endothelial metabolic-inflammatory crosstalk in SAE pathogenesis and position PTPRO inhibition as a promising, disease-modifying therapeutic strategy for this devastating complication of sepsis, where effective targeted interventions remain urgently needed.

## Supplementary Information

Supplementary Material 1.

## Data Availability

No datasets were generated or analysed during the current study.
