# Supplementary material for: Brain endothelial PTPRO drives LPS-induced metabolic reprogramming and neuroinflammation in sepsis-associated encephalopathy
Source: J Neuroinflammation. 2026 Mar 29;23:154. doi: 10.1186/s12974-026-03790-7 (PMC13154552; doi:10.1186/s12974-026-03790-7)
Supplement: Supplementary file 1 — Supplementary Material 1. [file 12974_2026_3790_MOESM1_ESM.docx]

**Supplementary Figure S1**

**Supplementary Figure S1. KEGG pathway analysis of LPS-induced transcriptomic changes in control (NC) and PTPRO-silenced (si-PTPRO) endothelial cells.** Pathways were ranked based on the magnitude of differences in enrichment p-values between NC and si-PTPRO cells. Top-ranked pathways preferentially activated by LPS in NC cells compared with si-PTPRO cells.

**Supplementary Figure S2**


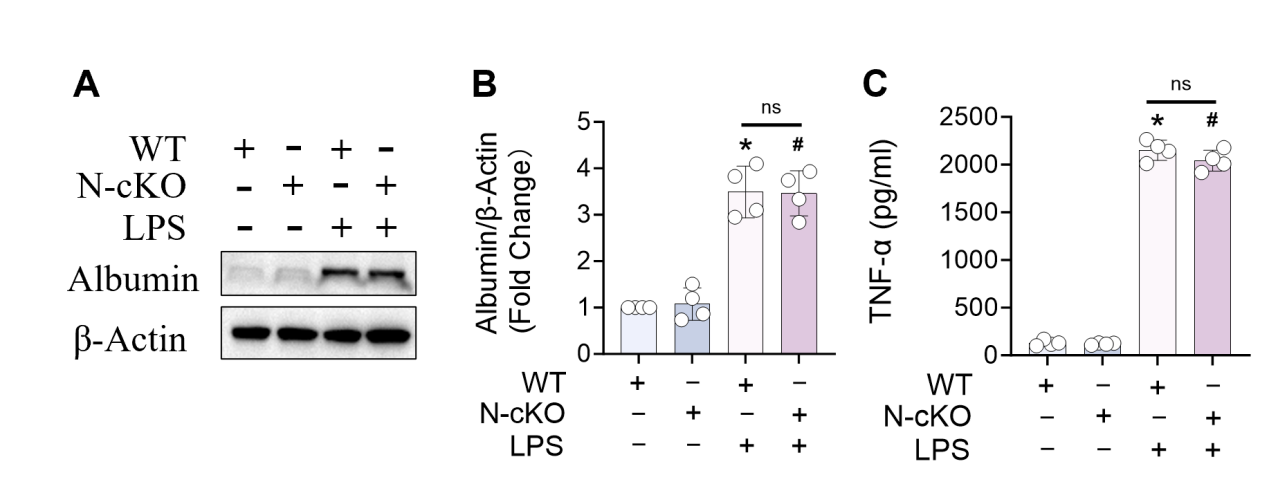


**Supplementary Figure S2. Neutrophil-specific PTPRO deficiency does not alter BBB permeability in LPS induced neuroinflammation.** Neutrophil-specific PTPRO conditional knockout mice (N-cKO) and wildtype mice (WT) were intraperitoneally injected with LPS (10 mg/kg) and analyzed 24 h later. (A-B) Representative immunoblot analysis and quantification of albumin levels in brain tissues (n = 4). (B) ELISA measurement of TNF-α protein levels in mouse brain tissues (n = 4). Data are presented as mean ± SD (n=4). *p < 0.05 vs WT control group; ^#^p < 0.05 vs N-cKO control group; ns, not significant.

**Supplementary Figure S3**


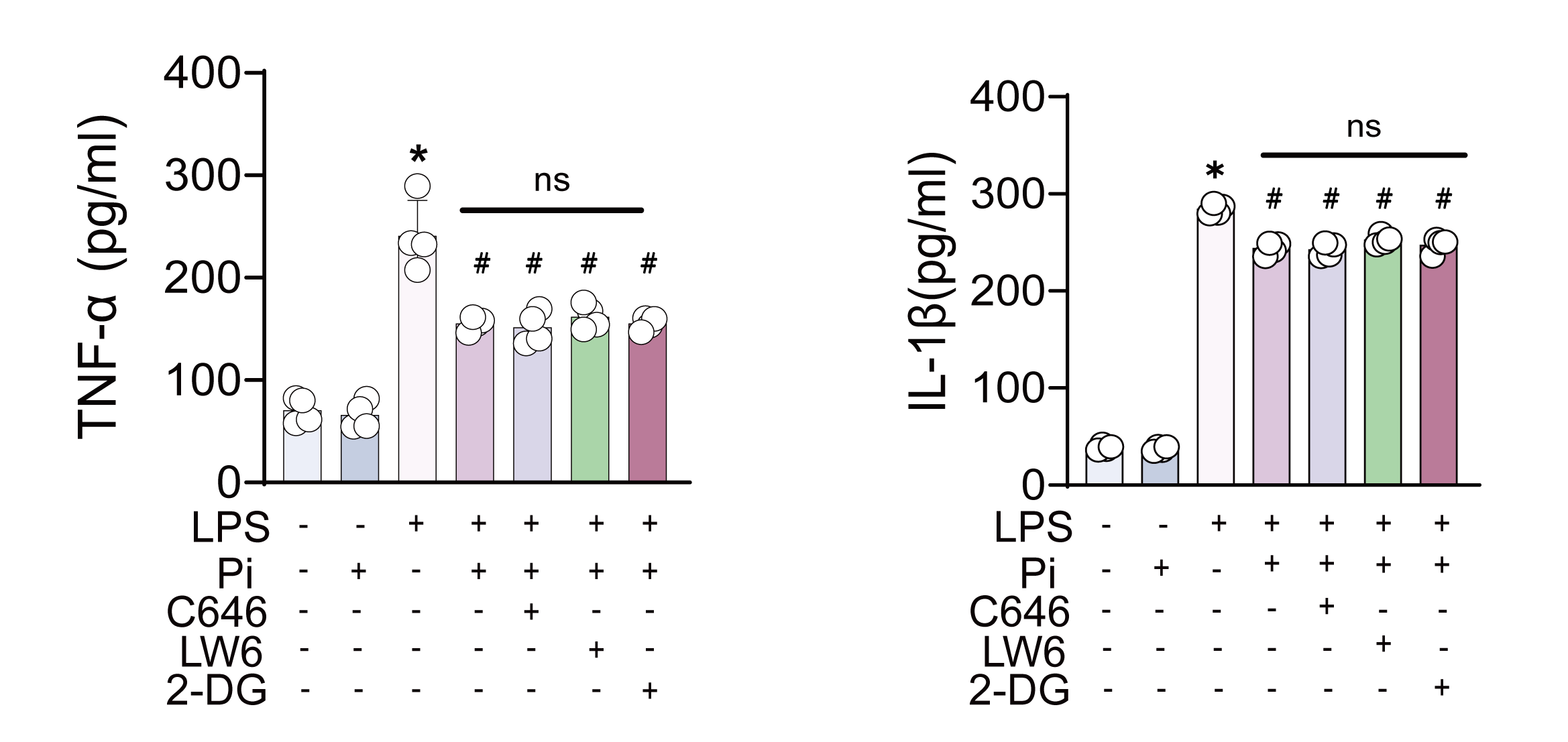


**Supplementary Figure S3. Combined inhibition of PTPRO and HIF-1α/glycolysis/lactylation fails to exert additive anti-inflammatory effects in LPS-stimulated brain endothelial cells.** Brain endothelial cells were stimulated with LPS and treated with the PTPRO inhibitor (Pi) alone or in combination with LW6, C646, or 2-DG. The levels of TNF-α and IL-1β in the culture supernatant were measured to evaluate inflammatory responses. Compared with the respective single-inhibitor groups, combined treatment with Pi + LW6, Pi + C646, or Pi + 2-DG did not result in a statistically significant further reduction in cytokine production. These findings indicate that inhibition of PTPRO, HIF-1α, glycolysis, and lactylation does not exert additive anti-inflammatory effects under LPS stimulation, supporting their involvement in a functionally connected pathway. Data are presented as mean ± SD. Statistical analysis was performed using one-way ANOVA followed by multiple-comparison testing. *p < 0.05 vs control group; ^#^p < 0.05 vs LPS-only group; ns, not significant.
